# Application of metabolomics in quality control of traditional Chinese medicines: a review

**DOI:** 10.3389/fpls.2024.1463666

**Published:** 2024-11-15

**Authors:** Peiran Ji, Xinquan Yang, Xiangsheng Zhao

**Affiliations:** ^1^ Institute of Medicinal Plant Development, Chinese Academy of Medical Sciences and Peking Union Medical College, Beijing, China; ^2^ Hainan Branch of the Institute of Medicinal Plant Development, Chinese Academy of Medical Sciences & Peking Union Medical College, Haikou, China

**Keywords:** Traditional Chinese medicine, metabolomics, quality, safety, multiomics

## Abstract

Plant metabolites are the components endowing traditional Chinese medicine (TCM) with therapeutic effects, and, simultaneously, they are the primary targets for quality control. From germplasm selection and origin determination to field management, growth duration, harvesting and processing, and, finally, storage and transportation, each step profoundly influences TCM quality. The complexity of these plant or herb metabolites poses numerous quality control challenges. Metabolomics, as a comprehensive and systematic approach, has demonstrated value in this field. This technique not only meets the requirements for studying the complex mechanisms of TCM but also has significant advantages in identifying the TCM components, including active components. Therefore, in this article, several key factors affecting the chemical characteristics and quality traits of TCM, including their origin, harvesting period, medicinal parts, and processing methods, are researched. Additionally, the current challenges of integrating metabolomics with other omics technologies (transcriptomics, spatial metabolomics, etc.) are discussed. Furthermore, a future development trends and prospects are highlighted. With the continuous deepening of research and ongoing updates in technological capabilities, metabolomics will play an increasingly important role in the quality control of TCM, providing more scientific and robust support for quality assurance and safety evaluation.

## 1 Introduction

Traditional Chinese medicine (TCM) has a thousand-year history in China and is widely employed for the treatment and prevention of diseases. Moreover, TCM is playing an increasingly crucial role in modern healthcare. With the gradual global recognition of TCM in recent years, the TCM industry has experienced rapid development in various countries. Compared with chemically synthesized drugs, Chinese herbal formulations consist of multiple natural herbs, each potentially containing hundreds or even thousands of active components. The variations in the combinations of different herbs and their sources further increase the chemical complexity. However, the quality control system for Chinese medicinal materials, which is the foundation of the TCM industry, still has some limitations. Although comprehensive monographs and standards, including definitions, characteristics, identification, testing, determination, and storage information, for TCM have been established worldwide and documented in publications such as pharmacopeias and directives, this information is insufficient for exerting comprehensive and effective control over factors influencing the quality of TCM (including germplasm, origin, field management, growth duration, harvesting, processing, storage, and transportation) (Commisso et al., 2013; Li, 2019). The subsequent pharmaceutical processes, involving extraction, purification, separation, concentration, and drying, significantly impact the quality of TCM. Currently, the regulatory framework for TCM quality control is meticulously structured around key elements. Firstly, it is firmly rooted in a comprehensive legal foundation, encompassing laws, regulations, and normative documents such as the Pharmaceutical Administration Law, the Law on Traditional Chinese Medicine, and related implementing regulations and measures. Building on this, the National Medical Products Administration (NMPA) has formulated Specialized Provisions for TCM Standards, providing strong legal backing. Secondly, the framework establishes a TCM-specific standard management system that covers various areas like medicinal materials, decoction pieces, formula granules, extracts, and proprietary medicines. This system ensures comprehensive management across national, registration, and provincial standards. Lastly, while preserving traditional knowledge and techniques, the framework actively integrates modern science and technology. It focuses on developing practical and economically reasonable TCM standards, thereby ensuring the scientific and feasible nature of the quality control system. At present, the quality control paradigm for TCM predominantly relies on the quantitation of active (or marker) ingredients and fingerprint technology. While these methodologies offer valuable insights into the content of bioactive compounds present in TCM, they often exhibit a certain degree of specificity, rendering a comprehensive and precise evaluation of the holistic quality of the herbs challenging. Consequently, they prove inadequate in providing an exhaustive and comprehensive depiction of key metabolites and their metabolic pathways (Li et al., 2018b; Munekata et al., 2022). In addition to existing national or multinational legal frameworks, ensuring the quality of TCM relies on powerful analytical tools to determine their chemical composition and active principles. These tools help identify potentially harmful substances and adulterants, ensuring the efficacy and safety of TCM and enhancing the quality standards and regulatory levels of TCM products.

**Figure 1:**
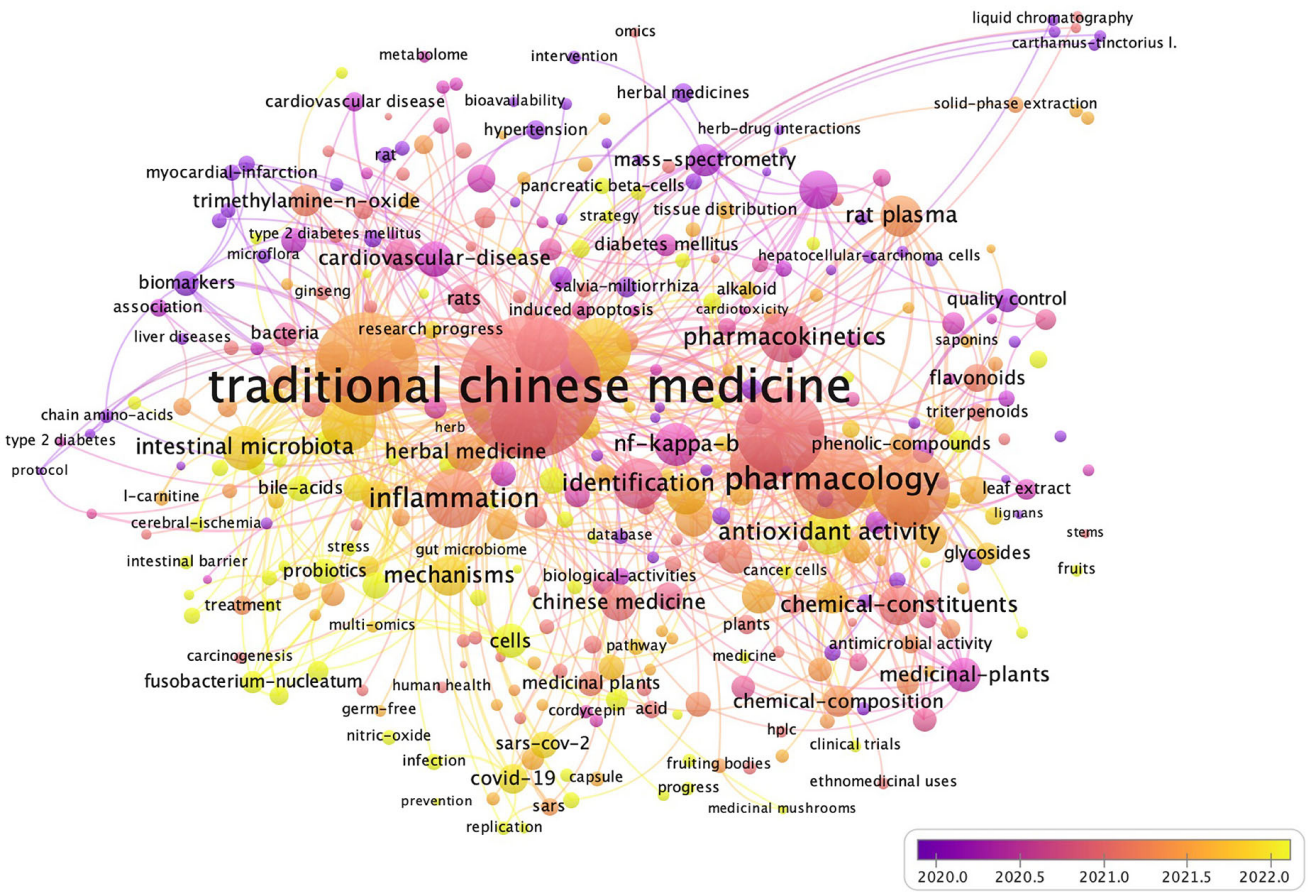
Analysis of research hotspots in the perspective of time (keyword co-occurrence frequency ≥2).

Metabolomics appears to be highly suitable for this purpose. As metabolism occurs, metabolites, which are small molecules involved in energy conversion and biosynthesis, are generated. External disturbances and developmental stimuli can impact the accumulation of intermediate or end products in plant cells or tissues over time and space. With the aim of capturing these changes, metabolomics technology monitors metabolites and their quantities in cells or tissues at a given state over time and space (Goodacre et al., 2004; Alawiye and Babalola, 2021). Furthermore, as the ultimate products of cellular regulation processes, metabolites serve as a bridge connecting the genotype and phenotype of plants. They provide crucial information about the phenotypic characteristics and biological functional changes in target organisms (Patti et al., 2012). For the quality control of TCM, metabolomics, leveraging its distinctive advantages of comprehensiveness and systematicness, enables a global analysis of small-molecule metabolites within TCM. It employs a variety of advanced separation and analysis technology platforms to precisely identify and accurately quantify characteristic metabolites, while integrating diversified data processing methods. This approach effectively avoids the limitation of relying solely on partial chemical components as quality control indicators, ensuring the comprehensiveness and accuracy of the evaluation. In recent years, beyond applications in clinical and biomedical fields, metabolomics research has been applied to various disciplines in food and nutritional science (Kang and Suh, 2022). Regarding research related to TCM, a literature survey based on the Web of Science core collection showed that in the last five years (from 2019 to the present of October 16, 2024), there have been 1998 publications on the theme of “traditional Chinese medicine” and “metabolites”, including 257 review papers (**Figure 1**). The core focus of these review papers is the practical application of the bioactive components of TCM in disease treatment (Zhang et al., 2021; Yang et al., 2020; Su et al., 2022; Zheng et al., 2020). Furthermore, several studies have investigated specific Chinese medicinal species (such as *Patriniae* (Gong et al., 2021), *Kaempferia galanga* L (Wang et al., 2021), and *Bufo bufo gargarizans* Cantor (Zhan et al., 2020)), genera (such as *Panax* L (Liu et al., 2020b), *Aconitum* [ (Kakkar et al., 2023), and *Asarum* (Liu and Wang, 2022)), and active ingredients (such as scoparone (Hui et al., 2020), rhaponticin (Chen et al., 2020a), and gentiopicroside (Liu et al., 2023a)), comprehensively elucidating their phytochemical, pharmacological, and pharmacokinetic characteristics. Moreover, advancements in the application of high-resolution mass spectrometry (HRMS) technology have been introduced (Yin et al., 2021; Huang et al., 2022), which holds significant importance in elucidating the efficacy and toxicity mechanisms of TCM (Bai et al., 2022). Some reviews have also assessed and analyzed the potential of secondary metabolites from medicinal plants for the treatment of the 2019 coronavirus (Jamal, 2022; Harwansh and Bahadur, 2022; Murck, 2020; Al-Kuraishy et al., 2022). However, a comprehensive review of the application of metabolomics technology in the quality control of TCM and the combined use of metabolomics with other omics methods to analyze various factors affecting the quality of TCM is still lacking. It is noteworthy that TCM, as an extensive and complex system, encompasses plant-based medicines, animal-based medicines, mineral-based medicines, as well as certain chemical and biological products. Among these, plant-based medicines undoubtedly occupy a pivotal position in the field of TCM, with a prominent proportion exceeding 80%. Therefore, the main content of this article primarily focuses on the application of metabolomics in the realm of plant-based medicines.

**Figure 2:**
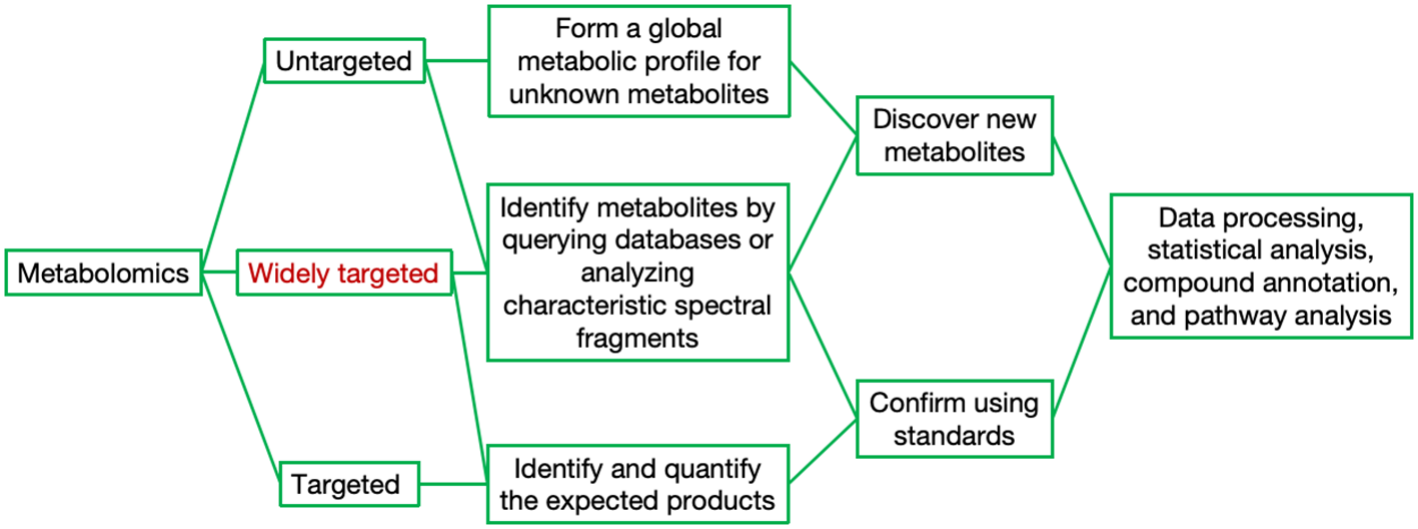
Targeted, widely-targeted, and untargeted approaches for metabolomic analysis.

Currently, out of the millions of potential metabolites in the plant kingdom, only approximately 14,000 can be practically quantified, which unequivocally underscores the pressing need for more advanced analytical techniques (Alseekh and Fernie, 2018). The hybrid approach that integrates both non-targeted and targeted metabolomics has demonstrated its efficacy in significantly enhancing the scope and depth of metabolome detection (**Figure 2**). In general, metabolomics can be divided into targeted metabolomics and untargeted metabolomics. Targeted metabolomics focuses on the identification and quantification of expected metabolites, requiring higher levels of purification and selective extraction of metabolites. In contrast, untargeted metabolomics aims to capture as many metabolites as possible without necessarily identifying or quantifying specific compounds (Sun et al., 2021; Schrimpe-Rutledge et al., 2016). In recent years, the novel metabolomic detection technique of widely targeted metabolomics, which integrates the advantages of the comprehensive nature of untargeted and the accuracy of targeted metabolomics, has been extensively applied, achieving high precision, sensitivity, rapid separation, and broad coverage (Wang et al., 2023a; Ren et al., 2023). The choice of separation and analytical platform varies depending on the sample type and research direction. Currently, commonly used platforms fall into two major categories: MS-based and nuclear magnetic resonance (NMR)-based platforms. The advantages and disadvantages of each platform are summarized in **Table 1**. Gas chromatography−mass spectrometry (GC−MS) is the most developed hybrid chromatography−MS technology widely applied in metabolomics research. The coupling of GC and MS offers several advantages, such as both instruments operating in the gas phase, direct connection, and a straightforward interface (Alawiye and Babalola, 2021; Fiehn, 2016; Xu and Feng, 2023; Liu and Locasale, 2017). Liquid chromatography−mass spectrometry (LC−MS) is also a commonly used analytical platform. In addition to having powerful separation capabilities, high sensitivity, and high resolution, LC–MS can overcome the limitations of GC−MS by effectively detecting nonvolatile, heat-sensitive, highly polar, and large-molecular-weight substances (Alawiye and Babalola, 2021; Liu and Locasale, 2017; Zhou and Yin, 2016; Yu et al., 2018). NMR has a limited dynamic range and lacks methods for determining the structures of unknown metabolites at low concentrations; hence, its application in plant metabolomics research is relatively limited (Alawiye and Babalola, 2021; Liu and Locasale, 2017; Emwas et al., 2019).

**Table 1:** Metabolomics platforms.

	Pros	cons
**GC-MS**	• High sensitivity and resolution • Suitable for natural volatile compounds and small molecule metabolites • Large-scale open-source database	• Thermally sensitive, non-volatile polar metabolites require derivatization • The molecular weight of the target analyte is limited • Low capacity • Destructive to the sample
**LC-MS**	• High sensitivity and resolution • Suitable for non-volatile, thermally unstable, and high-molecular-weight compounds • Broad detection range • Simple sample preparation • Superior flexibility in compound separation and detection, including the selection of LC columns, mobile phases, and MS method settings. • Large-scale open-source database	• Organic compounds that do not form molecular ion adducts (such as hydrocarbons) cannot be measured • Destructive to the sample • Matrix effect • Narrow linear range of response
**NMR**	• Simple sample preparation • Low sample consumption • Non-destructive • High-throughput, unbiased detection system • High reproducibility • Highly automated • Easy to perform real-time metabolite identification • Able to provide deeper structural information	• Narrow detection range • Low sensitivity • High equipment and maintenance costs

In recent years, metabolomics has undergone rapid development. Among its various techniques, Metabolic Flux Analysis (MFA) stands as a traditional yet potent method, utilizing stable isotope labeling experiments to delve into the metabolic pathways within biological systems. However, MFA encounters multiple constraints in practical applications, such as cellular heterogeneity, indirect analysis of energy and reducing power supplies, and the intricacies of experimental and analytical procedures. To circumvent these limitations, Flux Balance Analysis based on constraints (FBA) has emerged. FBA predicts flux distributions within metabolic networks under specified conditions through optimization techniques, offering a more flexible and robust alternative for exploring the metabolic capacity of plant metabolic networks (Kruger and Ratcliffe, 2021). Furthermore, single-cell metabolomics represents an emerging field that studies the composition and dynamic changes of metabolites within individual cells, encompassing advanced technologies like Laser Capture Microdissection, Laser Ablation Electrospray Ionization Mass Spectrometry (LAESI-MS), and pico Pressure Probe Electrospray Ionization Mass Spectrometry (picoPPESI-MS) (Katam et al., 2022). These techniques not only enhance our understanding of intercellular heterogeneity but also provide profound insights into metabolic pathways and cellular functions. For instance, by investigating the impact of UV-C radiation and melatonin treatment on secondary metabolite synthesis in *Lepidium sativum* L (Ullah et al., 2019), as well as exploring the effects of dolomite [CaMg(CO_3_)_2_] treatment on the growth, secondary metabolite production, and anti-malarial activity of *Sonchus arvensis* L. callus (Wahyuni et al., 2021), single-cell metabolomics offers a rich perspective and deep understanding of plant metabolism. Recently, researchers have developed a Widely Targeted Metabolite modificomics (WTMM) strategy. This strategy combines UHPLC-Q-Trap and UHPLC-QE-Orbitrap technologies, conducting systematic studies on metabolite modifications using tomato as a model. The WTMM strategy not only enables large-scale detection and quantitative analysis of modified metabolites in plants but also provides robust support for the development of plant biomarkers. This innovative research approach not only broadens the application scope of metabolomics but also furnishes new perspectives and tools for a deeper understanding of plant metabolic mechanisms (Yang et al., 2024). The represent cutting-edge technologies in the field of metabolomics. Regrettably, these advanced techniques have not yet been applied in the realm of quality control for TCM. Nevertheless, with the continuous deepening and expansion of research in these related fields, there is reason to believe that these sophisticated technologies will play a significant role in the quality control of TCM in the future.

Therefore, the focus of this review is to compile the latest advancements in metabolomics methods for assessing the quality and safety of TCM components. In this article, several key factors influencing the chemical profile and quality traits of TCM, including their origin, harvesting, and processing methods, are described. The current challenges of integrating metabolomics with other omics technologies, such as transcriptomics, have been actively explored in this field. Furthermore, future development trends and prospects are discussed.

## 2 Metabolomics for enhancing TCM quality

**Figure 3:**
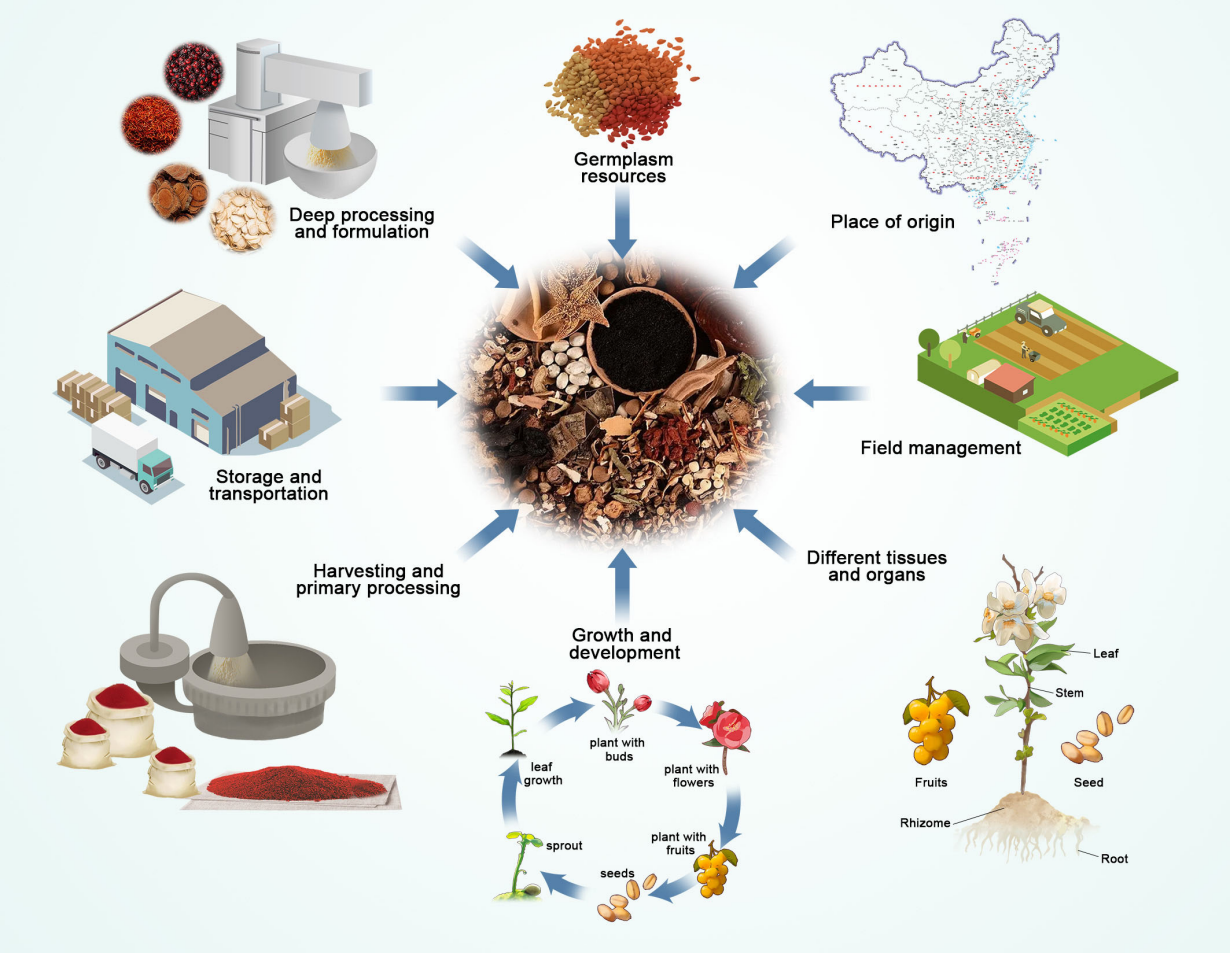
Factors affecting the quality and safety of TCM.

The quality of TCM is a crucial factor in determining its effectiveness and safety, and it has a direct impact on all subsequent steps of pharmaceutical production. The visual characteristics and active components of TCM are essential criteria for assessing their quality and are subject to various internal and external factors. From the selection of germplasm origins to the precision of field management, the application of harvesting and processing techniques, environmental conditions during storage and transportation, and the judicious selection of processing methods, each step can have an impact on the quality of TCM. Subsequently, various factors influencing the quality of TCM were detailed in this review, and the specific applications of metabolomics technology in controlling these factors to enhance the quality of TCM are discussed. Factors related to the quality of TCM are illustrated in **Figure 3**.

### 2.1 Origin

The origin of TCM is directly related to their quality, efficacy, and safety. The growth of plants in different environments and under different cultivation methods can result in variations in growth rate, yield, and active ingredients, leading to the unique regional characteristics of TCM. Although genuine medicinal materials have superior quality, their high price and demand have led to the presence of substitutes and adulterants on the market. Therefore, accurate identification is crucial for the quality control of TCM. Traditional identification methods are time-consuming and lack accuracy. Metabolomic techniques can be applied to the identification of TCM of different geographical origins, ensuring the quality and safety of the herbs and preventing their confusion and substitution (Tang et al., 2021).

In-depth research on the differences in the quality of Goji (*Lycium chinense* Miller) plants from different climatic regions was conducted by Yao et al (Yao et al., 2018). By observing morphological characteristics, they found that Goji plants cultivated in the monsoon region are lighter in weight, smaller in size, and shinier than those cultivated in other regions. On the other hand, Goji plants cultivated in the plateau region of Ningxia are the largest and brightest. However, despite the ability of ^1^H NMR technology to distinguish between the two species, there were no significant differences in the quality of Goji plants from different cultivation regions. Based on differences in origin and variety, *Fritillariae Bulbus* is classified into three major categories: Chuan-Beimu, Zhe-Beimu, and Ping-Beimu. Among them, Chuan-Beimu stands out for its outstanding therapeutic effects and low toxicity, resulting in a price several times higher than that of Zhe-Beimu and Ping-Beimu (Li et al., 2009). However, due to the complexity of the market, some businesses, in pursuit of profits, use low-priced varieties to impersonate Chuan-Beimu, severely impacting the quality of TCM and consumer rights (Luo et al., 2018). Liu et al., (2020a) successfully collected eight medicinal species of Chuan-Beimu (*Fritillariae Bulbus*), conducted comprehensive metabolic fingerprint analysis of Chuan-Beimu and its counterfeits using nontargeted metabolomics methods, and further applied chemometric methods to determine the characteristic substances of Chuan-Beimu and its counterfeits.

Gao et al. (2012) conducted a metabolomic analysis of the flower buds of seven species of Lonicera using LC-QTOF MS technology. By optimizing extraction conditions, chromatographic separation parameters, and mass spectrometry detection protocols, they achieved comprehensive detection of metabolites in the flower buds of these Lonicera species. Leveraging molecular feature extraction algorithms and multivariate statistical analysis methods, the researchers identified 82 statistically significant metabolite markers that exhibited notable distribution differences across the various Lonicera species. Through subsequent analysis of these markers’ chemical classes and fragment ion information, they successfully established a rapid and accurate method for discriminating among the flower buds of the seven Lonicera species. The metabolomics-based approach to the identification of TCM facilitates the prompt distinction between substitutes and adulterants, thereby not only contributing to the protection of consumer rights but also promoting the healthy development of the TCM market.

Although research on heavy metal and microbial contamination in the field of TCM currently appears to be relatively weak, and the main focus of studies has largely centered on areas related to staple crops, such as exploring how *Fusarium graminearum* affects the quality degradation of stored wheat (Niu et al., 2024), as well as the absorption characteristics and interaction mechanisms of sweet potato (*Ipomoea batatas* L.) with uranium (U) and cadmium (Cd) (Lai et al., 2020), with the continuous deepening and expansion of research in these related fields, these findings will undoubtedly inject new vitality into the quality control of TCM in the future and provide valuable references and insights.

Apart from the medicinal materials mentioned above, metabolomics technology is also widely applied in studying the quality differences of TCM from various sources and varieties. For instance, researchers have conducted in-depth studies on *Codonopsis lanceolata* from different production areas using metabolomics methods to explore quality differences and potential impacting factors (Nam et al., 2023). Furthermore, studies have focused on different varieties of Dandelions (Zhang et al., 2021), *Pulsatilla Adans* (Zhang et al., 2019), *Eucommia ulmoides* Oliv (Wu et al., 2018), and other TCM, exploring quality differences using metabolomics technology. These studies provide methods and perspectives for in-depth understanding of the metabolic differences and bioactivity of medicinal plants from different sources, contributing to better quality control and standardization of medicinal plants.

### 2.2 Growth and development

The medicinal components of TCM usually reach their optimal therapeutic efficacy and contain the highest levels of active ingredients during specific growth stages. Moreover, at certain growth stages, the plants exhibit the best visual qualities, including color, shape, and size. Selecting the appropriate harvest time is crucial for obtaining relatively high-quality TCM. Currently, there is a shortage of wild medicinal resources, and in the case of cultivated herbs, inadequate understanding of ecological environments, regional differences, human activities, and the growth patterns of medicinal herbs during actual harvesting leads to a decline in the quality of TCM (Chen et al., 2021; Liu et al., 2022). Metabolomic methods utilizing technologies such as MS and NMR can be used to thoroughly analyze small-molecule metabolites during the growth and development of TCM, revealing changes in their composition and content. This provides a powerful basis for scientifically determining the maturity of TCM.

*Eucommia ulmoides*, which is widely distributed in China, has significant applications in the medical field due to its high content of chlorogenic acid (CGA), flavonoids, lignans, and other compounds in its leaves and bark. In a recent study, Li et al. (2019) employed nontargeted metabolomics and chemometrics to analyze the metabolic fingerprint patterns of *Eucommia ulmoides* leaves at different growth stages and identified characteristic substances that can be used to accurately distinguish between different growth stages. Most flavonoid compounds reached their peak accumulation in growing leaves, followed by mature leaves. The present study also revealed a stable increase in the large accumulation of CGA during leaf growth and development, while rutin exhibited higher accumulation levels in leaf buds and growing leaves. Additionally, this study investigated the transcriptome of leaves at different growth stages and revealed dynamic changes in gene expression. In addition to leaves, floral medicinal materials exhibit significant diversity due to the synthesis and accumulation of pigments. During the growth of safflower (*Carthamus tinctorius* L.), the color of the flowers gradually transitions from yellow to red. Researchers such as Ren et al. (2022) conducted a comprehensive analysis of flavonoid biosynthesis in safflower flowers through metabolomics and transcriptomics, detecting changes in flavonoid compound biosynthesis on the 2nd day (yellow stage) and 4th day (red stage) of flowering. The authors identified a total of 212 different flavonoid metabolites, with the levels of hydroxysafflor yellow A and carthamin significantly increasing during the color transition, indicating their crucial role in changing flower color from yellow to red. Xia et al. (2021) conducted a metabolomic and transcriptomic analysis of *Lonicera japonica* flower petals at different developmental stages, employing nontargeted and targeted metabolomics to analyze metabolite changes during the green, white, and yellow stages. By combining these data with transcriptomic data, they extensively investigated gene expression changes involved in pigment accumulation. The results showed that flavonoid and carotenoid synthesis and accumulation played a critical role in the color transition of *Lonicera japonica* flower petals. By applying metabolomics techniques or integrating them with other omics approaches, researchers have investigated the differences in quality during different growth and development stages of TCM, such as *Scutellaria baicalensis* (Sun et al., 2023), *Fritillaria hupehensis* (Duan et al., 2023), and *Ganoderma lingzhi* (Satria et al., 2019; Xie et al., 2020). These research findings provide a scientific basis for optimizing the cultivation, harvesting, and subsequent development and utilization of TCM.

### 2.3 Medicinal parts

Medicinal herbs typically consist of multiple medicinal parts, such as roots, stems, leaves, flowers, fruits, and seeds. Each part may contain different medicinal components, allowing for the selection of the appropriate medicinal part based on the desired pharmacological effect. On the other hand, due to differences in the growth period and the content of medicinal components in different parts of medicinal herbs, utilizing the characteristics of various medicinal parts can maximize the resource utilization efficiency of these herbs. Liu et al (Liu et al., 2017), using nontargeted metabolomics methods, conducted a comprehensive analysis of the metabolite profiles in different tissues, including roots, stems and leaves of *P. ginseng* and *P. quinquefolius*. By comparing the metabolite compositions of different tissues, researchers identified several significantly different metabolites, mainly amino acids, organic acids, sugars, and other primary metabolites. Furthermore, researchers have performed correlation analyses between the metabolite profiles of different tissues and the accumulation of saponin components. The results showed a close association between the changes in primary metabolites and the accumulation of saponin components in different tissue parts. For example, in the roots and leaves of *P. ginseng*, the contents of amino acid metabolites such as glycine and serine were positively correlated with the accumulation of ginsenosides. In the roots and stems of *P. quinquefolius*, the contents of organic acid metabolites such as malic acid and citric acid were negatively correlated with the accumulation of *P. quinquefolius* saponins. Chen et al. (2020b) reported that the content of saponins in the stems and leaves of *P. ginseng* and *P. quinquefolius* was significantly greater than that in the roots and flowers. In a combined metabolomics and glycomic analysis of the xylem and cortex of *Morinda officinalis* Radix (MOR), researchers found that, compared to the cortex, the xylem contained more potentially toxic components, such as vernolic acid, physcion and linoleic acid. However, the xylem has relatively few bioactive components, such as rubiadin-1-methyl ether (Yip et al., 2019). Cui et al. (2018), using GC−MS technology, conducted an in-depth analysis of the metabolites in the rhizomes, flowers, leaves, and stems of *Fritillaria thunbergii* Miq. Through multivariate data analysis, the authors found significant differences in primary metabolites in the rhizomes compared to the other three plant parts. Metabolites in the rhizomes formed one cluster, while those in the flowers, leaves, and stems formed another cluster. Li et al. (2022) also conducted a nontargeted metabolomics study on the rhizomes and flowers of *Fritillaria thunbergii* Miq., and their results indicated substantial chemical composition differences between the two plant parts. Some alkaloids and flavonoids were found to be more abundant in the flowers than in the rhizomes. Molecular network analysis categorized the obtained metabolites into two main groups, with one group mainly present in the rhizomes and the other enriched in the flowers. The chemical differences among different plant parts may affect the efficacy and mechanisms of action of *Fritillaria thunbergii* Miq. Therefore, in drug development and clinical applications, it is essential to consider the chemical composition differences among different plant parts to maximize the pharmacological effects of medicinal herbs. Metabolomic techniques have also been applied to study quality differences among different medicinal plant parts, such as *Codonopsis pilosula* (Zeng et al., 2021), *Lonicera japonica* Thunb (Wang et al., 2023c), and velvet antler (Tseng et al., 2014).

Plant medicines are typically complex systems composed of multiple organs and tissues (Dong et al., 2020). In traditional metabolomic studies, researchers often crush and homogenize plant samples, but this approach can lead to the loss of valuable spatial information (Li et al., 2023; Dai et al., 2020). Spatial information is crucial for understanding metabolic processes, as various metabolites continuously interact in complex and delicate ways within plant tissues. Fortunately, the emergence of spatial metabolomics has provided an effective solution to this problem. Spatial metabolomics is a new field that integrates mass spectrometry imaging (MSI) technology with metabolomics. It can simultaneously analyze the spatial distribution characteristics of hundreds of metabolites on the same tissue section and utilize high-resolution mass spectrometry to deeply analyze differential components in the region, thereby closely linking dynamic changes in metabolites with spatial distribution (Heyman and Dubery, 2016). MALDI-MSI currently stands as the most extensively utilized MSI technique. The process begins with the placement of the sample within a carrier gas chamber, where a laser serves as a dependable ionization source. Following this, the sample is subjected to raster scanning, a methodical procedure that systematically captures mass spectrum data from various locations. By integrating the intensity of ion signals with the XY coordinates of the sample’s surface, it is possible to generate detailed ion maps or mass spectrometry images (Lange et al., 2017). These high-resolution maps not only afford precise determination of the molecular weights and structural attributes of compounds but also offer valuable insights into their distribution patterns and concentration gradients within the sample. These high-resolution maps not only afford precise determination of the molecular weights and structural attributes of compounds but also offer valuable insights into their distribution patterns and concentration gradients within the sample (Norris and Caprioli, 2013; Lange et al., 2017). In contrast, DESI-MSI employs charged fine droplet beams that are rapidly sprayed onto the sample surface. These droplets efficiently extract and dissolve the analytes. As the solvent evaporates swiftly, charges are transferred from the droplets to the analyte molecules, facilitating the desorption and ionization of the molecules on the sample surface. Concurrently, with the aid of gases such as nitrogen, the charged sample droplets undergo desolvation and are directed along the ion transfer tube into the mass spectrometry detector for analysis. A significant advantage of DESI technology lies in its elimination of the need for sample pretreatment, enabling analysis to be conducted at atmospheric pressure (Yuan et al., 2024; Wang et al., 2023b). Lastly, SIMS-MSI holds the distinction of being the MSI technique with the highest spatial resolution. It utilizes a focused primary ion beam to strike the sample surface, resulting in the sputtering of positive and negative secondary ions (Heyman and Dubery, 2016). Through spatial metabolomics technology, we can intuitively observe the distribution of specific plant metabolites at different growth stages and in various organs, revealing the patterns of metabolite changes over time and space. This makes the intricate secondary metabolic pathways of plants clear and greatly advances our understanding and research on the complex system of plant medicines (Li et al., 2013; Sun et al., 2020). Studies indicated that metabolites that accumulate in the roots of *Tripterygium wilfordii* exhibit considerable toxicity, necessitating spatial isolation to prevent adverse effects (Brinker et al., 2007). Using MALDI-MSI, researchers have assessed the localization of metabolites. The results revealed that two major quinone methide triterpenoids, celastrol and demethylzeylasteral, predominantly accumulated in the periderm tissue. Researchers speculated that this accumulation pattern might be related to *Tripterygium wilfordii*’s defense against bacteria and/or fungal soil pathogens. Additionally, they found via MSI that triptophenolide, a diterpenoid, accumulated significantly more in the woody roots of *Tripterygium wilfordii* and was slightly enriched in the periderm. Conversely, sesquiterpene alkaloids showed a more even distribution, primarily concentrated in the root cortex (Dai et al., 2020). Leveraging MALDI-MSI, Kuo et al. (2019) successfully obtained mass spectral images of agarwood samples, revealing the distribution of chemical components in different regions and providing an intuitive visual reference for subsequent studies. By associating MSI data with known compounds and utilizing molecular network analysis and machine learning algorithms, they successfully inferred the possible structures of new compounds. After experimental verification and structural elucidation, the structures and properties of the new compounds were ultimately confirmed. Furthermore, researchers have used MALDI-MSI technology to conduct metabolomic studies on the roots of two plants in the genus *Paeonia*, *Paeonia suffruticosa* and *Paeonia lactiflora*. The results showed significant spatial heterogeneity in the distribution of metabolites in these plant roots, indicating differences in metabolic activities in different regions of the roots (Li et al., 2021). Tong et al. (2022) and other researchers have studied the distribution and compositional characteristics of metabolites in different tissue parts of *Salvia miltiorrhiza* by combining metabolomics with DESI-MSI. The authors inferred the biosynthetic pathways of *S. miltiorrhiza* through data analysis and corresponding studies. The results demonstrated a close correlation between the distribution and composition of metabolites in different tissues of *S. miltiorrhiza* and the associated biosynthetic pathways.

Spatial metabolomics has addressed the limitations of traditional metabolomics in *in-situ* visualization analysis, thus introducing a fresh perspective and dynamism to the field of quality control in TCM. Nonetheless, several challenges persist in its practical application. Specifically, there is a lack of standardized operational procedures and quantitative methodologies, necessitating the establishment of unified guidelines. Furthermore, there is an urgent demand for novel, high-sensitivity mass spectrometry imaging devices capable of balancing high resolution with rapid imaging speeds.

### 2.4 Processing and formulation

Processing refers to a series of procedures, including drying, frying, steaming, and roasting, etc., applied to TCM, and this process is a distinctive feature of TCM. Through processing, the efficacy of medicinal materials can be enhanced, toxicity can be reduced, and taste and aroma can be improved. Chemical components in TCM inevitably undergo changes during these processes. In terms of quality control, the assessment of processed product quality often relies on the quantitative analysis of a few active ingredients. However, since TCM comprise complex components, evaluating its overall quality based on the analysis of a few components alone is challenging (Kui et al., 2022). By employing metabolomic approaches to comprehensively analyze substances undergoing changes during processing, detect trends in the alteration of medicinal components, and understand the biological transformation pathways under different processing conditions, it is possible to optimize the processing techniques regarding their impact on the medicinal components of TCM.

*Cymbopogon citratus* (citronella grass), a plant use for fragrance development and as a traditional herbal medicine, can be more easily stored and transported after drying than when fresh (Oladeji et al., 2019). However, the differences in medicinal components between dried and fresh citronella grass are currently unclear. To address this issue, Xiong et al. (2023) conducted chemical component identification and determination of fresh and dried citronella grass and explored the changes in metabolite concentrations before and after drying. Citronella grass is rich in volatile and nonvolatile components, including flavonoids, amino acids, organic acids, and vitamins. The contents of flavonoids and certain amino acids increased, while the contents of organic acids, other amino acids, and vitamins decreased during the drying process. Notably, flavonoids have beneficial effects, such as antioxidant properties (Figueirinha et al., 2008) and cardiovascular protection (Formica and Regelson, 1995), and positively influence human health. Additionally, drying altered the astringency of citronella grass, making it more suitable for medicinal use. Kang et al. (2021), utilizing UHPLC-Q/TOF-MS/MS, conducted a study on the overall chemical composition and differences between *Morinda officinalis* Radix (MOR) and processed *Morinda officinalis* Radix (PMOR). In this study, they successfully analyzed 41 batches of MOR samples and 32 batches of PMOR samples, identifying a total of 110 common components. The results indicated significant differences in the contents of 55 compounds between MOR and PMOR. Specifically, 29 components, including fructooligosaccharides, monotropein, deacetylasperulosidic acid, geniposide, and anthraquinone glycosides, had relatively high concentrations in MOR. On the other hand, PMOR had higher concentrations of 26 components than MOR, with difructose anhydrides and iridoid glycoside derivatives being discovered in PMOR for the first time. In a study investigating the chemical composition changes of processed Moutan Cortex, Li et al. (2018a) analyzed 30 samples using ESI-Q/TOF-MS/MS. These samples included 11 batches of raw Moutan Cortex (RMC), 9 batches of Moutan Cortex Tostus (MCT), and 10 batches of Moutan Cortex Carbonisatus (MCC). Through screening in negative ion mode, 14 chemical markers were successfully identified. These markers included monoterpene glycosides, acetophenones, gallic derivatives, flavonoids and carbohydrates. Importantly, most chemical markers exhibited a significant decrease after frying of MCC. Specifically, the levels of monoterpenoid glycosides, such as oxypaeoniflorin, decreased markedly after processing, potentially leading to a decrease in its antithrombotic effect. Moreover, the content of dissociated gallic acid increased after frying, suggesting that gallic acid may enhance the anti-inflammatory and hemostatic functions of Moutan Cortex. These research findings provide a deeper understanding of the compounds and therapeutic changes that occur during the processing of TCM, offering robust support for the modernization and standardization of these materials.

In addition to the abovementioned TCM, differences between raw and processed Radix Rehmanniae (Li et al., 2010) and *Atractylodes macrocephala* Koidz (Shan et al., 2014) have also been investigated. Metabolomic techniques have been used to study the overall components changes during processing, such as the effect of boiling time on the overall quality of White Paeony Root (Ming et al., 2017), screening differentially abundant metabolites in *Polygoni Multiflora* Radix with varying processing times (Yu et al., 2017) and revealing the overall chemical changes and the correlation of key markers during the distillation process of *Ligustri Lucidi* Fructus (Li et al., 2020). Furthermore, exploring the synergistic effects and detoxification of processing products, such as raw *Pinelliae Rhizoma* and processed *Pinelliae Rhizoma* with alum (Sun et al., 2019), could constitute another avenue for further investigations.

## 3 Multiomics technologies in TCM quality and safety

**Figure 4:**
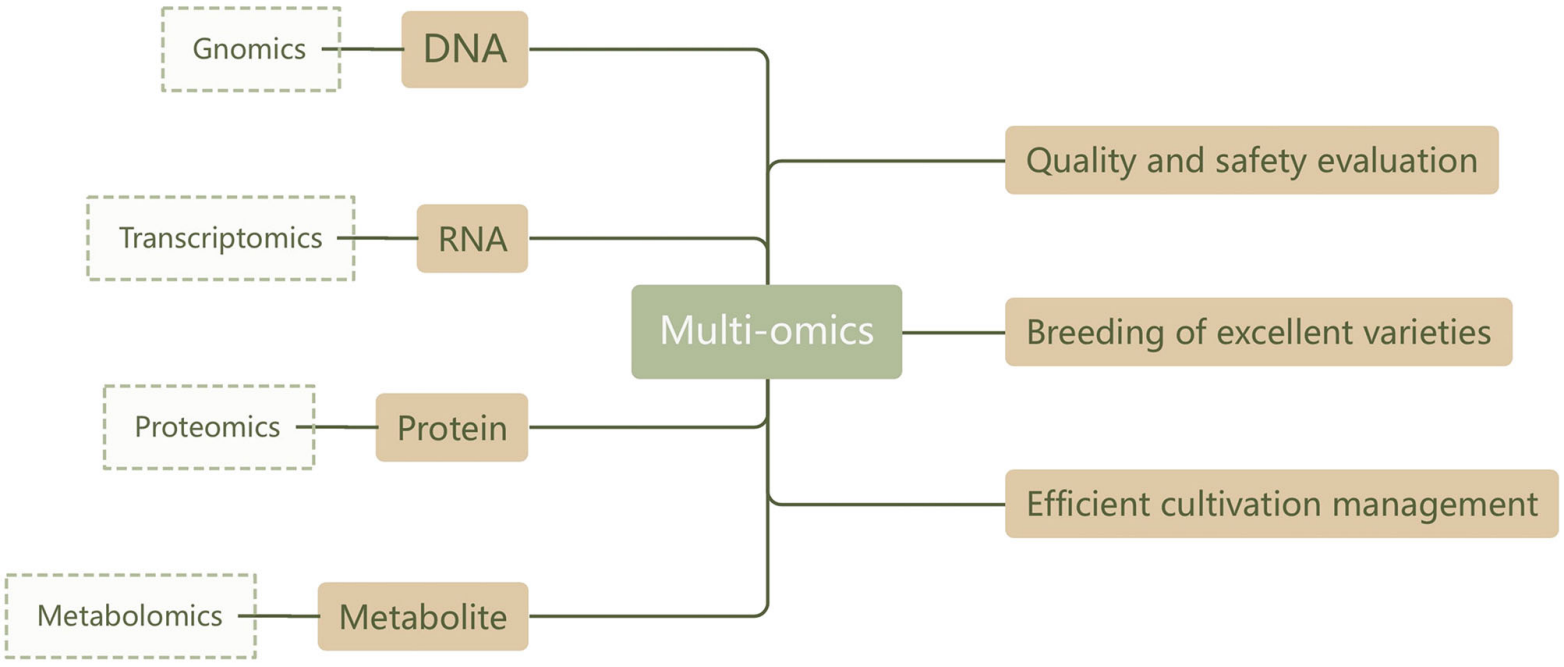
Technical route of multi-omics research.

Metabolomics analysis has demonstrated high accuracy and reliability in assessing the quality and safety of TCM. The advantage of this method lies in the fact that metabolites, as the end products of cell signaling, can directly reflect the organism’s immediate state and functional changes. This opens a window for observing and evaluating the condition of the organism (or product). However, relying solely on metabolomics cannot provide a complete understanding of the root causes of TCM quality, especially for botanical drugs. Their metabolic networks involve complex and diverse regulatory mechanisms that exist not only on the surface of metabolism (such as various tissue forms formed through protein-protein interactions) but also throughout the multi-gene, multi-level regulatory networks of the transcriptome and even epigenetics (Muthamilarasan et al., 2019; Ideker et al., 2001) Therefore, current metabolomics research is gradually trending towards integration with other omics fields (such as genomics, transcriptomics, and proteomics), aiming to comprehensively depict various functions from the genome to the metabolome, and then to phenotypic characteristics. This forms a more complete and in-depth understanding framework (Sheth and Thaker, 2014; Yang et al., 2021). The specific technical flowchart is shown in **Figure 4**.

As mentioned above, the combined analysis of metabolomics and transcriptomics (Li et al., 2019; Ren et al., 2022; Xia et al., 2021) is currently one of the most developed techniques in multiomics studies. This involves separately conducting transcriptome sequencing and metabolite detection of specific samples during a particular period. The data obtained from the transcriptome, which reveals numerous differentially expressed genes, is then correlated with the differentially abundant metabolites identified through metabolomic analysis. This correlation analysis aims to analyze the intrinsic changes in the organism at both the causative and resultant levels, identifying pathways enriched with genes and metabolites. The two omics disciplines mutually validate each other, providing a comprehensive explanation of biological questions (Gahlan et al., 2012; Hao et al., 2011, 2012; Desgagné-Penix et al., 2012; Hagel et al., 2015). For instance, to delve into the alkaloid biosynthesis pathway of *Veratrum mengtzeanum*, researchers have employed transcriptomics and metabolomics to systematically analyze its various tissues (roots, stems, and leaves). Transcriptomic analysis revealed genes expressed in different tissues, including key genes closely related to alkaloid synthesis. Metabolomics identified various alkaloid components in different tissues. The results indicated that alkaloid biosynthesis in *V. mengtzeanum* mainly occurs in the roots and is closely related to specific transcript expression patterns. Despite earlier findings that only the roots were the most abundant source of alkaloid biosynthesis, this study revealed that both leaves and roots play crucial roles in alkaloid biosynthesis. This finding expands the medicinal efficacy of different *V. mengtzeanum* parts, providing important evidence for further exploration of its medicinal value (Liu et al., 2023b). Tanshinones in *Salvia miltiorrhiza* (Danshen) are important active components, which mainly accumulate in the periderm, giving the roots of Danshen their characteristic red color. However, during long-term cultivation, researchers have observed a variation in which the roots of Danshen turn orange. A comparative analysis of metabolites in orange and red Danshen roots revealed a total of 40 lipophilic components, among which 7 were significantly reduced in the orange variant, including the abundant active compounds tanshinone IIA and tanshinone I. Further gene expression studies showed that dehydrogenase-related gene expression was not downregulated, but genes related to stress resistance and endoplasmic reticulum (ER)-associated protein degradation were upregulated. This suggests that the dehydrogenase enzymes may not have decreased, but their functionality might be compromised. Investigations uncovered that these changes could potentially lead to misfolding of the catalytic C_15_-C_16_ dehydrogenase, which subsequently undergoes ER-associated degradation, resulting in a decrease of dehydrogenated furan ring tanshinones. This decrease is not solely caused by reduced gene expression. This reduction further affects the dehydrogenation conversion of other tanshinones, negatively impacting the overall quality of cultivated Danshen and causing the root color to shift from red to orange. Thus, this series of complex biochemical processes, rather than a single reduction in gene expression, is the key factor contributing to the decline in the quality of Danshen (Zhan et al., 2019). Genome-wide association study (GWAS) is a method to analyze genetic markers in large-scale population DNA samples at the genome-wide level, aiming to explore genetic factors associated with specific phenotypes, such as metabolite abundance. When GWAS is combined with metabolomics technology (i.e., mGWAS), it can deeply analyze the genetic mechanism of metabolite synthesis, thereby locking the gene loci that control metabolic synthesis and regulation (Duan et al., 2016). Through in-depth research on *Salvia miltiorrhiza*, scholars have compared the gene expression patterns of different diterpene synthesis pathways and their corresponding metabolites, thereby clarifying the importance of diterpene synthases (diTPSs). In their investigation of five Copalyl Diphosphate Synthases (SmCPSs), they observed that SmCPS3 may not be active, while the other SmCPSs correspond to different diterpene synthesis pathways. Interestingly, despite their biochemical similarity, SmCPS1 and SmCPS2 play distinctly different roles in the biosynthesis of tanshinones. This similarity is not meaningless but rather reflects their unique functions in different plant tissues (roots and aerial tissues). This novel discovery provides insights into increasing the yield of tanshinones in aerial parts, implying that we can sustainably and environmentally friendly obtain tanshinones by selectively cultivating and harvesting only the aerial tissues of the plant, without the need to destroy the entire plant (Cui et al., 2015). Regarding the joint analysis of metabolome and proteome, in the study of *S. baicalensis* mentioned in Section 2.2 (Growth and Development), researchers conducted an in-depth analysis of metabolite accumulation and protein expression changes in *S. baicalensis* at different growth years (i.e., “Zi Qin” and “Ku Qin”) through the combined application of untargeted metabolomics technology and label-free proteomics methods (Sun et al., 2023). The results have shown that there are differences in metabolite and protein expression of *S. baicalensis* at different growth stages, and these differences mainly focus on the biosynthetic pathways of phenylalanine, tyrosine, tryptophan, and their related compounds. These findings reveal the variation pattern of metabolism and protein expression of *S. baicalensis* with growth time, providing a strong basis for scientifically determining the optimal harvest time. Given the diversity and dynamic changes of secondary metabolites during the development of *Lonicera japonica* flowers (honeysuckle) (Jiang et al., 2009), coupled with the diversification of gene families involved in the phenylpropanoid synthesis pathway in honeysuckle and its closely related species, this uncertainty has a profound impact on the medicinal value of honeysuckle and its related species (Yuan et al., 2014; Fraser and Chapple, 2011). To delve deeper into this issue, Yang et al. integrated transcriptomics, proteomics, and metabolomics approaches to systematically dissect the tight relationship between molecular changes and the regulation of secondary metabolites during the development of honeysuckle flowers (Yang et al., 2019). After a comprehensive analysis of omics data, the research team found that the metabolic changes of honeysuckle during its developmental stages mainly focus on core pathways such as sugar metabolism, lipopolysaccharide synthesis, carbon conversion, and secondary metabolism. Interestingly, as the flower develops, the number of genes associated with cellular and secondary metabolism decreases, revealing dynamic gene expression during the development of honeysuckle. In-depth proteomics research indicates significant differences in protein expression between the early and late stages of flower development. In the early stages, proteins related to glycolysis and phenylpropanoid metabolism are predominantly active, whereas in the later stages, proteins related to the citric acid cycle and terpenoid backbone pathway dominate. Epigenomics focuses on mapping various epigenetic regulators across the entire genome, exploring chemical modifications of the genome (such as DNA methylation, histone modifications, etc.) and changes in spatial structure without altering the DNA sequence. It is worth noting that the various physiological processes experienced by medicinal plants during growth and development are profoundly influenced by epigenetic marks, which directly or indirectly regulate various life activities of plants (He et al., 2011; Guo et al., 2021). Given the important role of DNA methylation and secondary metabolism in medicinal plants’ response to cold stress, researchers have explored the effects of cold exposure time on American ginseng (Hao et al., 2020). They observed American ginseng at different growth stages and under various durations of cold treatment, examining the accumulation of ginsenosides, DNA methylation levels, and gene expression. The study revealed that DNA methylation and demethylation in perennial American ginseng dynamically adjust in response to seasonal temperature changes. Adequate cold exposure during winter can facilitate sufficient DNA demethylation in early spring tender leaves, subsequently promoting the high expression of specific genes during flowering and fruiting stages, and ultimately leading to the maximum accumulation of ginsenosides in the roots of American ginseng. Ginsenosides are the core medicinal components of American ginseng, and their content directly affects the efficacy and quality of the medicinal material. This study not only provides a scientific basis for the quality control of American ginseng but also suggests that environmental factors should be fully considered in the quality control of TCM to ensure the optimal quality.

Despite the significant potential demonstrated by multi-omics approaches in the field of quality control of TCM, their application still faces multiple challenges and limitations. The primary difficulty lies in the complexity and analytical challenges of data processing, which stem from both the mismatch between the high-efficiency processing demands of massive data and current technological capabilities, as well as the complexity of integrating and analyzing data from different omics. This poses higher requirements for researchers’ interdisciplinary knowledge and the application of bioinformatics tools. Furthermore, there are limitations in sensitivity and resolution at the technological application level, particularly in the detection of low-abundance metabolites and proteins. Coupled with the challenge of ensuring the representativeness of TCM samples, this further exacerbates the uncertainty of the research. Additionally, the high research costs and time-consuming experimental processes constitute economic and temporal barriers to practical application. Nevertheless, with the continuous development of the combined application of metabolomics and other omics, we have been able to construct a panoramic multi-dimensional information platform through the organic integration of various omics technologies, including genomics, epigenomics, transcriptomics, proteomics, metabolomics, and phenomics. This platform is dedicated to deeply mining functional genes and comprehensively and systematically elucidating the synthesis and metabolic mechanisms of active components in TCM, thereby providing a more scientific basis for research on the quality control of TCM.

## 4 Conclusion and perspectives for the future

This review provides a thorough exploration of the utilization of metabolomics for ensuring the quality of TCM, delving into the essential elements that shape its overall superiority. These factors encompass various aspects, such as geographical origins, growth and developmental stages, medicinal parts, and processing methods, providing comprehensive theoretical support for the quality control of TCM. Furthermore, this review briefly introduces the application of spatial metabolomics visualization techniques in the study of metabolite distribution in medicinal plants, revealing the specific distribution of metabolites in different spatial contexts within plants. Although multiomics applications in this field are still in their early stages, their potential and prospects in the quality control of TCM have garnered widespread attention and discussion.

Although the application of metabolomics technologies in the field of TCM quality control is gradually expanding, the limitations and challenges it faces are becoming apparent, given the current technological landscape. First, the processing, interpretation, and validation of metabolomics data pose challenges. Due to the high-dimensional and multifaceted nature of metabolomics data, complex methods are required for statistical and bioinformatics analyses. However, different statistical methods and hypothesis settings may lead to disparate results, making the interpretation and validation of results challenging. Second, the identification of metabolite markers, especially unknown metabolites, requires further analysis. Many substances found in metabolomics lack standard spectra in current mass spectrometry databases, and acquiring corresponding standard substances is difficult, making substance identification a formidable task. Finally, metabolomics research faces issues of data reproducibility. Due to various interferences, such as differences in sample preparation and environmental factors, metabolomics data from the same sample may exhibit considerable variations across different laboratories and time points. Therefore, while metabolomics technologies hold immense potential in the quality control of TCM, overcoming these difficulties and challenges is essential. Continuous improvement and refinement of technology are necessary to enhance the accuracy of data analysis and the reliability of results.

In the future, the application of metabolomics research in the quality control of TCM may move forward in three key directions. Firstly, with the continuous improvement of separation and detection technology, metabolomics techniques will be able to detect and quantify more metabolites more accurately, including those present at low concentrations. Metabolomics research will tend towards automation, standardization, and integrity to improve data reliability and reproducibility. Secondly, metabolomics will be more tightly integrated with other omics data such as genomics, transcriptomics, proteomics, etc., to comprehensively elaborate the metabolic pathways and genetic structures of medicinal plants. This integration will help discover new biomarkers and understand the regulatory mechanisms of complex metabolic networks in medicinal plants. Finally, an important development direction will be to build and improve metabolite databases, especially for unique metabolites of TCM. This will assist researchers in more rapidly identifying unknown metabolites and understanding their functions and mechanisms of action in TCM.

With the continuous advancement of research technologies, the refinement of platforms, ongoing progress in genomics and transcriptomics, and the efficient application of artificial intelligence (AI) algorithms, particularly machine learning for handling large datasets, we believe that methods based on metabolomics will play an increasingly important role in research on the quality and safety assessment of TCM.
